# Functional correlates of self-reported energy levels in the Health, Aging and Body Composition Study

**DOI:** 10.1007/s40520-021-01788-0

**Published:** 2021-03-10

**Authors:** Rebecca Ehrenkranz, Andrea L. Rosso, Briana N. Sprague, Qu Tian, Theresa Gmelin, Nicolaas Bohnen, Eleanor M. Simonsick, Nancy W. Glynn, Caterina Rosano

**Affiliations:** 1 Department of Epidemiology, Graduate School of Public Health, University of Pittsburgh, Pittsburgh, PA USA; 2 Intramural Research Program, National Institute on Aging, Baltimore, MD USA; 3 Department of Neurology, University of Michigan, Ann Arbor, MI USA

**Keywords:** Energy, Fatigue, Successful aging, Epidemiology

## Abstract

**Background:**

Effects of fatigue on health in older age are well studied, yet little is known about the clinical relevance of energy perception.

**Aims:**

To explore cross-sectional associations of self-reported energy with physical and mental health metrics in the Health, Aging, and Body Composition Study.

**Methods:**

Participants rated their energy from 0 to 10; the outcome was energy dichotomized at the median (≥ 7 = higher energy). Four domains were assessed: depressive symptoms (Center for Epidemiologic Studies Depression Scale); physical performance (function: usual and rapid gait speed; fitness: 400-m walk time); physical activity (casual walking, walking for exercise, and intense exercise); and cognitive function (Modified Mini-Mental State Examination and Digit Symbol Substitution Test). Covariates bivariately associated with energy entered a multivariable logistic regression model, adjusted for demographics, chronic conditions, and strength.

**Results:**

Depressive symptoms, physical performance and activity, but not cognition, were bivariately associated with energy (*p* < 0.0005). Younger age, male sex, greater strength, and absence of chronic conditions predicted higher energy (*p* < 0.001). In a multivariable model, depressive symptoms [adjusted odds ratio (aOR) 95% CI 0.69 (0.62, 0.76)] and 400-m walk times [aOR = 0.81 (0.72, 0.91)] were inversely associated with energy; usual and rapid gait speed [aOR = 1.3 (1.2, 1.4); aOR = 1.2 (1.1–1.4)], and time spent in intense exercise [aOR = 1.4 (1.1–1.7)] were positively associated with energy.

**Discussion:**

In this cohort with a range of chronic conditions and fatigue, perceiving higher energy levels may reflect better emotional and physical health.

**Conclusion:**

Energy should be considered in multidimensional clinical assessments of older age.

**Supplementary Information:**

The online version contains supplementary material available at 10.1007/s40520-021-01788-0.

## Introduction

Consequences of aging are often studied and framed in relation to deleterious health outcomes, but less is known about the clinical relevance of maintaining higher levels of energy later in life. Recent research suggests that one’s perception of his or her energy level may be an indicator of global health status [1–3]. Reporting higher levels of energy may also reflect general life satisfaction and continued social participation [3, 4]. To date, little research describes the physical and mental health characteristics associated with higher levels of energy in older adults. Notably, fatigue, defined as lack of energy, is commonly evaluated in aging studies and its negative association with health outcomes have been reported [5–8]. However, the absence of fatigue does not necessarily affirm the presence of higher energy levels.

Energy and fatigue are often viewed as opposites on a continuum, yet there is increasing evidence they have different neurobiological correlates [9–11]. For example, recent research suggests alterations in dopamine are associated with changes in energy but not fatigue [9]. Conversely, increased serotonin levels have been linked to fatigue but not to energy [9, 12]. In a small double-blind placebo controlled trial in participants under age 40, those given a histamine precursor reported reduced fatigue with no changes in energy [9, 13]. These results indicate that energy and associated health characteristics should be evaluated separately from fatigue.

Energy can be measured objectively or subjectively. Objective measures primarily capture traits or physical fitness [14–16]; whereas, subjective measures capture energy state as well as perception of that state. Emerging evidence indicates subjective measures and individual perceptions are important predictors of clinically relevant outcomes [17–19]. For example, those who self-report favorable perceptions of aging had faster walking speed, better memory, and longer life span than those with unfavorable perceptions of aging [18, 20–22]. Analogously, self-reported energy may be associated with similar health-relevant characteristics.

In this study, we examined older adults’ objective measures of physical and mental health in relation to self-reported energy levels in a cohort of community-dwelling men and women with a range of chronic conditions. We hypothesized that lower depressive symptoms, higher physical performance, higher levels of fitness and physical activity, and better cognitive function would be associated with higher self-reported energy.

## Methods

The Health, Aging and Body Composition (Health ABC) Study is a longitudinal cohort study that enrolled 3075 Black and White community-dwelling adults aged 70–79 between March 1997 and July 1998 with the overarching objective to evaluate risk factors and disparities in the onset of functional limitation in healthy older adults. To be eligible, participants at baseline had to be free of reported difficulty walking ¼ mile or climbing 10 steps [23]. Health ABC provides the opportunity to examine both singular and composite associations between common, related factors (such as body mass index) and energy expenditure and fatigue.

In the current analyses, we selected those who answered the question about self-reported energy over the past month at Year Three (calendar years 1999–2000) of the study. All other variables are derived from Year Three, except for time to walk 400 m (assessed at Year Two) and the following variables ascertained at baseline: race, sex, education, peripheral artery disease, and Digit Symbol Substitution Test (DSST) results. Arthritis, cancer, diabetes, and cardiovascular conditions were assessed annually, and prevalence of these conditions was calculated at Year 3.

### Self-reported energy

Self-reported energy, the main outcome, was captured as follows: “Using this card, please choose the category that best describes your usual energy level in the past month on a scale of 0 to 10 where 0 is no energy and 10 is the most energy that you have ever had.”

### Main independent variables: physical and mental characteristics of interest

Depressive symptomatology was assessed using the Center for Epidemiologic Studies Depression Scale Revised 10 (CESD-10) questionnaire [24]. CESD-10 scores ≥ 11 indicate clinical depressive symptoms, scores between 5 and 10 indicate subclinical depressive symptoms, and scores < 5 indicate normal mood [25]. A modified CESD-10 score was computed for sensitivity analysis after exclusion of two questions that could be interpreted as related to energy and fatigue: “I felt that everything I did was an effort” and “I cannot get going” [26].

Physical performance was captured with measures of physical function and physical fitness. Physical function was measured as usual and rapid-pace gait speed (meters/second) during a 20-m walk at Year 3. Physical fitness was assessed as the time it took a participant to walk 400 m as quickly as possible, with longer walk times consistent with lower fitness [27]; a total of *n* = 1715 (68% of the sample) completed this test.

Physical activity was measured by asking participants about their walking habits in the prior week, and their answers were converted to total minutes of walking per week. Participants were also asked if over the last 12 months they did any of the following at least 10 times: walked for exercise, walked to complete day-to-day tasks, climbed a flight of stairs, and engaged in high-intensity exercise; answers were recorded as yes/no.

Cognitive function was assessed via the Modified Mini-Mental State (3MS) Examination and Digit Symbol Substitution Test. The 3MS is a screening test for cognitive impairment with a score range of 0–100, with higher scores indicating better cognitive function [28]. 3MS score cut-offs for clinical disorders are scores < 80 points, subclinical disorder cut-offs are 80–85 points, and normal status scores are > 85 [25, 29]. The DSST is a neuropsychiatric test of psychomotor performance which involves matching symbols to numbers [30]. DSST score is the total number of accurate symbol–number matches made in 90 s [25, 30].

### Fatigue and other health-related measures of interest

Fatigue was captured by asking the question: “In the past month, on average, have you been feeling unusually tired during the day?” Response options were yes or no, and if yes, participants reported if they felt unusually tired: all of the time, most of the time, or some of the time. Responses were grouped as yes or no for the analysis.

Demographic variables included chronological age, sex, race and highest level of education (all collected via self-report). Prevalent, pre-existing chronic conditions included: cardiovascular disease, diabetes, cancer, peripheral arterial disease, and osteoarthritis. These were measured via self-report questionnaires, medications listed, or Health Care Finance Administration diagnosis. A composite “cardiovascular conditions” variable was created to capture the presence of any of the following: coronary disease, chronic heart failure, cardiovascular disease, or hypertension. Anthropometric measures included: body mass index (BMI, measured as kg/m^2^] and quadriceps strength, measured as peak torque results from Kin-Com dynamometer [31].

### Statistical analyses

In this cross-sectional analysis, energy was the dependent variable; depressive symptoms, physical performance and activity, and cognition were the main independent variables. Self-reported energy was skewed towards higher scores and dichotomized at the median into higher and lower energy (Supplemental Figure 1). Given that participants may have interpreted the energy question differently based on personality or other factors, dichotomizing self-reported energy served to minimize potential variation and may improve the likelihood that those in the higher energy group truly had greater self-reported energy than those in the lower energy group. *T* tests or Chi-square tests were used to evaluate the associations of continuous or categorical variables with energy, respectively.

When significant (*p* < 0.05), bivariate associations for the main independent variables were found, we included these variables in multivariable logistic regression models with energy level as the outcome. Each main predictor entered one regression model at a time, with further adjustment for age and sex and further adjustment for covariates. Selection of covariates was based on bivariate associations with self-reported energy at *p* < 0.05. All main independent measures associated with energy entered a final combined model. Continuous independent variables were in standardized units.

Sensitivity analyses assessed whether results differed when including the following variables in the models: (a) fatigue (b) a modified CES-D score, excluding the two energy-related questions; (b) clinical cut-offs for CESD-10 (normal: scores < 5, subclinical depressive symptoms: scores 5–10, and clinically depressive symptoms: scores ≥ 11); (c) clinical cut-offs for 3MS (normal: scores > 85, impaired: scores ≤ 85); (d) clinical cut-offs of usual gait speed (normal: speed ≥ 1.0 m/sec, impaired: 0.6 < speed < 1.0 m/sec, disabled: speed ≤ 0.6 m/sec); (e) data from those who completed the 400 m walk; that is, all models were repeated in the subsample of *n* = 1715.

## Results

Participants were aged 76 ± 2.8 years, with an even distribution of men and women, a slightly higher proportion of Whites than Blacks, and less than half with post-secondary education (Table 1). Older participants, women, and those with less education were more likely to be missing the energy variable (*p* < 0.05).

**Table 1:** Sample characteristics and associations with higher and lower self-reported energy in the Health, Aging and Body Composition Study (*N* = 2529)

Characteristic	Total sample	Higher energy	Lower energy	*p* value
	*N* = 2529 Mean ± SD or N (%)	*N* = 1462 Mean ± SD or N (%)	*N* = 1067 Mean ± SD or N (%)	
Demographics				
Age in years	75.6 ± 2.8	75.4 ± 2.8	75.9 ± 2.9	0.0002
Men	1,223 (48.4)	744 (50.9)	479 (44.9)	0.003
White	1,555 (61.5)	878 (60.1)	677 (63.5)	0.08
Post-secondary education	1,121 (44.5)	655 (44.9)	466 (43.8)	0.6
Depressive symptoms and fatigue				
CESD-10^a^ score	7.6 ± 3.1	7.0 ± 2.6	8.3 ± 3.5	< 0.0001
Modified CESD-10 score^*^	6.8 ± 2.5	6.5 ± 2.2	7.1 ± 2.8	< 0.0001
Any unusual tiredness	621 (24.6)	199 (13.6)	422 (40.0)	< 0.0001
Physical function				
Usual gait speed, m/sec	1.15 ± 0.22	1.18 ± 0.21	1.10 ± 0.22	< 0.0001
Rapid gait speed, m/sec	1.52 ± 0.34	1.56 ± 0.33	1.46 ± 0.34	< 0.0001
Physical fitness				
Time to walk 400 m, m/sec^b^	328.6 ± 60.4	321 ± 59.4	339.6 ± 60.3	< 0.0001
Physical activity				
Walking for exercise (past 12 months, ≥ 10 times)	1,655 (65.4)	998 (68.5)	654 (61.4)	< 0.0001
High-intensity exercise (past 12 months, ≥ 10 times)	671 (26.6)	426 (29.2)	244 (22.9)	< 0.0001
Time spent walking weekly, minutes	102.8 ± 175	113.6 ± 183	88.1 ± 163	0.0003
Cognitive function				
Teng 3MS^c^	89.9 ± 8.8	90.0 ± 8.5	90.0 ± 8.9	0.9
Digit symbol substitution score	36.6 ± 14.3	36.61 ± 14.9	36.61 ± 13.6	0.9
Pre-existing health conditions				
Diabetes, prevalent	443 (17.5)	232 (15.9)	211 (19.8)	0.01
Cancer, ever	541 (21.5)	291 (20.0)	250 (23.5)	0.034
Cardiovascular conditions, prevalent^d^	1560 (61.7)	856 (58.6)	704 (66.0)	< 0.0001
Peripheral arterial disease, prevalent	120 (4.9)	49 (3.4)	71 (6.9)	< 0.0001
Arthritis, prevalent	969 (38.3)	498 (34.1)	471 (44.1)	< 0.0001
Anthropometric measures				
Peak torque (mean ± SD)	78.0 ± 29.1	80.3 ± 28.9	74.3 ± 28.9	< 0.0001
Body mass index, kg/m2 (mean ± SD)	27.2 ± 4.8	27.1 ± 4.7	27.3 ± 4.8	0.4

Distributions of participant characteristics by energy group. *T* tests were used for continuous variables, and Chi-square tests for categorical variables

^a^Center for Epidemiologic Studies Depression Scale 10 * Without two questions: “I felt that everything I did was an effort,” and “I could not get going”

^b^Time to walk 400 m was only recorded for 1,715 participants who completed the walk successfully

^c^Teng Mini-Mental State Exam

^d^Cardiovascular disease was a composite variable including: coronary heart disease, chronic heart failure, cardiovascular disease, and hypertension

Observed self-reported energy ranged from the lowest possible score of 0 to the highest possible score of 10 and 58% were categorized as having higher energy (Table 1). Prevalence of pre-existing chronic conditions such as composite cardiovascular disease (61.7%) and cancer (21.5%) was similar to what is observed in community-based samples of this age [32–34]. Similarly, average values of depressive symptoms, physical and cognitive function were as expected for community-dwelling adults in this age group [25, 35, 36]. Around 7% had clinically depressive symptoms, 20% had cognitive impairment based on 3MS score, and 24% had a gait speed < 1 m/sec. More than half of the sample reported walking for exercise (65.4%, *n* = 1655) and around a quarter of the sample reported undertaking high-intensity exercise (26.6%, *n* = 671).

### Results of bivariate associations

Fewer depressive symptoms, higher physical function and activity were bivariately associated with higher energy at *p* < 0.001 (Table 1). Associations were similar for the modified CESD-10 score that excludes the fatigue-related items. Younger age, being male, having higher muscle strength, and presence of any pre-existing chronic conditions except cancer were also bivariately associated with higher energy at *p* < 0.05 (Table 1). Although higher energy and lower fatigue (unusual tiredness) were significantly negatively correlated (correlation coefficient = -0.33, *p* < 0.001), a relatively high proportion of those reporting higher energy also reported fatigue (40%).

### Results of separate multivariable logistic regression models predicting higher energy

The association of CESD-10 score with higher energy was independent of all covariates. For each standard deviation increase in CESD-10 score, there was approximately a 25% lower odds of reporting higher energy in fully adjusted models [aOR = 0.69 (0.62–0.76)]. Results were similar across all model iterations (Table 2); results were attenuated when using the modified CES-D [aOR = 0.84 (0.76–0.92)] that excludes the fatigue-related items.

**Table 2:** Odds ratios and 95% confidence intervals of separate multivariable logistic regression models of mood and physical function associated with higher energy in the Health, Aging and Body Composition Study (*N* = 2529)

Independent variable^a^	Unadjusted	Adjusted for age and sex	Further adjusted for pre-existing chronic conditions^b^	Further adjusted for muscle strength
Depressive symptoms	0.65 (0.60–0.71)	0.66 (0.61–0.72)	0.68 (0.62–0.75)	0.69 (0.62–0.76)
Usual walk speed	1.5 (1.3–1.6)	1.4 (1.3–1.5)	1.4 (1.2–1.5)	1.3 (1.2–1.4)
Rapid walk speed	1.4 (1.3–1.5)	1.4 (1.3–1.5)	1.3 (1.2–1.4)	1.2 (1.1–1.3)
Time to walk 400m^c^	0.72 (0.65–0.80)	0.74 (0.66–0.82)	0.76 (0.68–0.84)	0.80 (0.71–0.91)
Minutes walking per week	1.2 (1.1–1.3)	1.1 (1.0–1.2)	1.1 (1.0–1.2)	1.1 (0.99–1.2)
Walking for exercise	1.4 (1.2–1.6)	1.3 (1.1–1.6)	1.3 (1.1–1.5)	1.3 (1.0–1.5)
High intensity exercise	1.4 (1.2–1.7)	1.4 (1.1–1.6)	1.4 (1.1–1.6)	1.4 (1.1–1.7)

Odds ratios for each independent variable in progressive model iterations. Depressive symptoms, usual and rapid walk speed, time to walk 400 m, and high-intensity exercise were significantly associated with higher energy in each iteration

^a^Each row reports results of separate models, where the variable listed in the first column is the main independent variable, and the model is progressively adjusted for covariates that were bivariately associated with energy. Note: the main independent variables are in standardized units

^b^Adjusted for: peripheral artery disease, diabetes, cardiovascular disease, cancer, arthritis

^c^Time to walk 400 m was recorded for 1715 participants who completed the walk successfully

All measures of physical function (performance and fitness) were associated with higher energy. Faster usual and rapid gait speed over 20 m were associated with greater odds of higher self-reported energy [aOR = 1.3 (1.2–1.5) and aOR = 1.2 (1.1–1.4) in fully adjusted models, respectively, or 30–20% difference for each standard deviation of the independent variable]. Longer time to walk 400 m was associated with lower odds of reporting higher energy in fully adjusted models [aOR = 0.81 (0.72–0.91)], or a 20% difference for each standard deviation of the independent variable. Results were significant in all model iterations (Table 2).

More physical activity was associated with higher energy, with stronger associations for walking for exercise and engaging in high-intensity exercise (30–40% difference for each standard deviation of the independent variable) compared to walking to complete day-to-day tasks (about a 10% difference for each standard deviation of the independent variable).

Neither measure of cognitive function was associated with energy in either unadjusted or adjusted models. The odds ratios for both remained at or below 1.0 as covariates were progressively added to each model (not shown). None of the interactions tested were significant (*p* > 0.1 for all).

### Results of the final multivariable logistic regression model predicting higher energy and sensitivity analyses

**Fig. 1:**
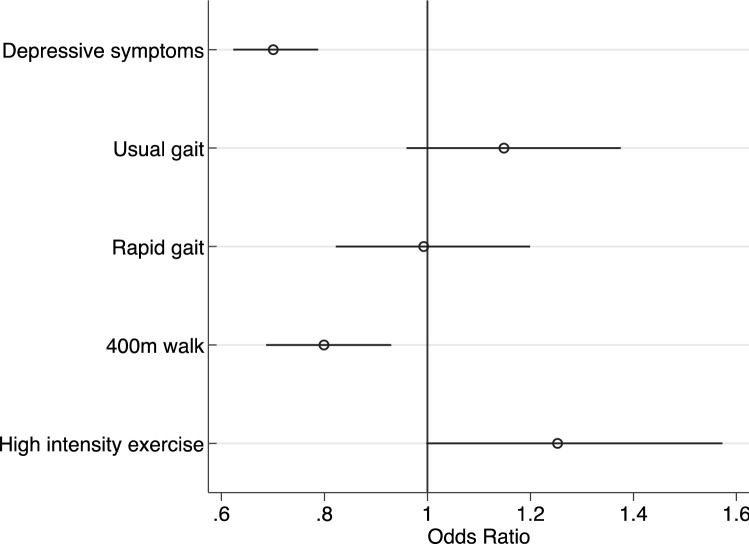
Correlates of higher self-reported energy in the Health, Aging and Body Composition Study (*N* = 1715). Figure legend: In a model including all significant correlates of higher energy (depressive symptoms, usual and rapid gait, 400-m walk times, and high-intensity exercise), depressive symptoms, 400-m walk time, and intense exercise remained associated with higher self-reported energy

A final model to evaluate higher self-reported energy included all independent variables that were associated with energy in separate multivariable models described above. Of note, this model was limited to the subgroup of those who completed the 400-m walk (subsample of *n* = 1715). In this model, only CESD-10, 400-m walk time, and time spent engaged in high-intensity exercise remained associated with energy (Fig. 1). In this model, the coefficients of age, cardiovascular disease, arthritis, and muscle strength were also statistically significant (Supplemental Table 1). Results were similar when using the modified CES-D (not shown).

Sensitivity analyses controlling for fatigue yielded similar results for each model (not shown). Using clinical cut-offs for CESD-10 (with all questions included) and no depressive symptoms as the reference category yielded results where subclinical depressive scores were not significantly associated with energy [aOR = 0.95 (0.75–1.2)], but clinically depressive scores were strongly associated with energy [aOR = 0.42 (0.30–0.57)]. Clinical cut-off categories for 3MS did not yield significant results in either bivariate or multivariable models.

## Discussion

Our results suggest that self-reported energy may reflect a person’s emotional and physical health (depressive symptoms, gait speed and fitness), as well as the extent to which a person would make use of his or her capacity in everyday life (walking and other exercise). These associations were robust to adjustment for other energy-related factors, including demographic characteristics, pre-existing chronic conditions, muscle strength, and fatigue. This analysis addresses a gap in our understanding of fatigue, energy and health characteristics in older age, by examining associations between objective cognitive and physical performance metrics and energy perception. Our data indicate that energy and fatigue are not entirely overlapping domains. In this sample, among those who reported higher energy, 40% also reported unusual tiredness over the last month, indicating that one is not simply the absence of the other. This distinction may be because energy and fatigue states are driven by different underlying neurobiological mechanisms [9].

Our results that depressive symptoms are inversely related with energy is consistent with prior work in clinical and patients’ populations. Reporting feelings of low energy are common symptoms of depressive disorders, especially in older age [36–39]. For example, one study found that decreased activity levels and increased apathy (defined as lack of motivation and affective dullness) was present in 75% of older depressed individuals [40]. Sensitivity analyses with clinical cut-off indicated associations are driven by more severe depressive symptoms. Of note, results were only partially attenuated when using the modified CESD-10, which excluded fatigue-related items. Although depressive symptoms are correlated with increased risk of reduced functional ability [41–43], accurate early detection of depression remains challenging, especially for older adults who do not openly disclose their emotional status to their physician. Future studies should examine whether reporting lower energy may be an accurate indicator of underlying depressive symptoms.

Our analyses advance our understanding of the link between physical function and energy. Much of the prior research on energy and gait has focused on objective measures of energy expenditure [40, 44, 45], but the results of this analysis indicate that gait speed has also robust associations with self-reported higher energy in the context of healthy aging. The 400 m walk can discriminate walking ability and fitness and can be used to identify early signs of functional decline in older adults [46]. Taken together, our results suggest that physical capacity is a distinct phenotypic characteristic of older adults reporting higher energy levels.

Across metrics of physical activity, those who reported exercising were more likely to also report higher self-reported energy than those who did not exercise. This relationship was not significant for those who reported engaging in day-to-day physical activity (such as walking to complete basic tasks or climbing stairs). It has been demonstrated that exercise can increase energy levels, and exercise has multiple mechanisms of action in the central nervous system that may directly affect energy perception [9, 47, 48]. Physical activity levels may be predictive of improved perception of higher energy level and may signal quality of life status in aging populations [44, 49]. These dose–response relationships parallel the results presented here, wherein higher levels of physical activity had the strongest associations with higher self-reported energy.

Contrary to our hypothesis, neither the 3MS nor the DSST was significantly associated with self-reported energy. There is little available literature on the connection of either of these two tests to energy. Reports of cognitive function tests and fatigue are often focused on vulnerable population subgroups (such as cancer patients or Parkinson’s patients) and thus, results may not be applicable to healthy older adults. The 3MS may not be the most appropriate test to capture the range of cognitive abilities in well-functioning populations due to the ceiling effect [50, 51]. It is possible that self-reported energy is related to other cognitive domains that are only partially captured by these tests, such as attention, inhibition, or task-switching.

### Limitations and strengths

Conceptualizations of energy may differ between participants, leading to variability between individual energy score rankings; we tried to temperate this limitation by dichotomizing the values at the median value. The trade-off is that by dichotomizing we may have lost statistical power to detect a difference where one exists. Self-reported fatigue was asked right before self-reported energy, which may have influenced participants to report lower than actual energy. This potential misclassification may have biased results towards the null if it made those reporting lower energy more similar on characteristics of interest to those reporting higher energy. Objective metrics of energy expenditure were not available. This was a cross-sectional evaluation of self-reported energy, so temporality and evaluating longitudinal changes in energy were beyond the scope of this analysis. As this was a healthy and relatively active cohort, results may be more generalizable to healthy older adults rather than the entire population of older adults. Of note, within this sample, those who were able to complete the 400 m walk may have had better overall health than those who were not. Other limitations pertain to the assessment of physical activity. Participants were asked about their levels of activity over the past year and may not accurately remember variations in day-to-day activity; participants may also have been more likely to recollect intentional exercise than time spent walking for general purposes. If so, results for high-intensity exercise may be biased away from the null. Additionally, participants were asked about their physical activity over multiple fairly similar questions, and their answers and accuracy may have varied depending on which questions were more meaningful to the individual participant.

A strength of this study is that it included numerous functional, performance-based, clinical and laboratory measures, including information on fatigue, as well as disease states. While there is substantial literature elucidating relationships between fatigue and aging, this analysis fills a gap by examining associations between objective cognitive and physical performance metrics and energy perception. For example, in this sample, among those who reported higher energy, 40% also reported unusual tiredness over the last month, indicating that one is not simply the absence of the other. These results build upon recent findings that there is a relationship between self-reported energy and physical activity by both objective and subjective measures [44]. Additionally, results from the Health ABC cohort are generalizable to well-functioning older adults living independently in the community. Numerous clinical measures were available, including information on both disease states and body composition. While it was not unexpected that pre-existing chronic conditions were associated with lower energy, we also noted a lack of significant association for the anthropometric measures BMI and muscle strength. This may indicate that body composition does not reflect energy in the way that physical function and activity do.

## Conclusion

Our results indicate that when reporting on their recent energy levels, older adults may be referring to physical performance and emotional wellbeing, independent of pre-existing chronic conditions. This has direct relevance to the psychosocial framework of aging, as self-reported energy may be indicative of underlying mood and physical characteristics that can either reduce or improve quality of life. By including self-reported energy as part of multidimensional clinical assessments in older adult populations, there may be opportunities to develop new interventions around modifiable behaviors that can lead to increased energy and lower risk for diminished functional capacity. Directions for future research may include evaluating self-reported energy as a predictor of functional impairment or mortality, and should include longitudinal measures of energy perception in older adults.

## Supplementary Information

Below is the link to the electronic supplementary material.

Supplementary file1 (DOC 72 KB)

## Data Availability

Data are publicly available; see the Health ABC website, Analysis Proposals section: https://healthabc.nia.nih.gov/analysis-proposals-publications.
