# Supplementary material for: Functional correlates of self-reported energy levels in the Health, Aging and Body Composition Study
Source: Aging Clin Exp Res. 2021 Mar 10;33(10):2787–95. doi: 10.1007/s40520-021-01788-0 (PMC8531104; doi:10.1007/s40520-021-01788-0)
Supplement: Supplementary file 1 — Supplementary file1 (DOC 72 KB) [file 40520_2021_1788_MOESM1_ESM.doc]

***Supplemental Table 1. Odds ratios and 95% confidence intervals of multivariable logistic regression models associated with higher energy in Health, Aging and Body Composition Study***

| **Independent**  **variables:^1^** | **Adjusted for CES-D10^2^** | **Adjusted for Usual Gait Speed** | **Adjusted for Rapid Gait Speed** | **Adjusted for Fitness** | **Adjusted for Physical Activity^3^** | **Adjusted for All Significant Correlates** | |  |
| --- | --- | --- | --- | --- | --- | --- | --- | --- |
|  |
| Age | 0.88 (0.80 - 0.97) | 0.90 (0.82 – 0.99) | 0.89 (0.81 - 0.98) | 0.90 (0.80 – 1.0) | 0.87 (0.80 – 0.96) | | 0.92 (0.82 – 1.0) |  |
| Sex | 1.0 (0.82 – 1.3) | 1.0 (0.80 – 1.3) | 1.0 (0.82 - 1.3) | 1.1 (0.87 - 1.5) | 0.97 (0.78 - 1.2) | | 1.2 (0.91 - 1.6) |  |
| Peripheral artery disease | 0.75 (0.52 – 1.1) | 0.71 (0.50 - 1.0) | 0.71 (0.49 - 1.0) | 0.71 (0.43 – 1.2) | 0.68 (0.48 - 0.98) | | 0.77 (0.46 - 1.3) |  |
| Diabetes | 0.82 (0.64 - 1.0) | 0.84 (0.66 - 1.1) | 0.82 (0.64 - 1.0) | 0.76 (0.56 - 1.0) | 0.79 (0.62 – 1.0) | | 0.80 (0.59 - 1.1) |  |
| Cardiovascular disease | 0.83 (0.69 – 1.0) | 0.86 (0.71 - 1.0) | 0.84 (0.70 – 1.0) | 0.80 (0.64 – 1.0) | 0.82 (0.68 - 0.99) | | 0.83 (0.66 - 1.0) |  |
| Arthritis | 0.75 (0.62 - 0.90) | 0.73 (0.61 - 0.89) | 0.73 (0.61 - 0.89) | 0.80 (0.64 – 1.0) | 0.71 (0.59 - 0.86) | | 0.83 (0.66 – 1.0) |  |
| Cancer | 0.74 (0.59 – 0.92) | 0.71 (0.57 – 0.89) | 0.71 (0.57 – 0.88) | 0.62 (0.48 – 0.80) | 0.73 (0.58 – 0.90) | | 0.64 (0.49 – 0.83) |  |
| Peak torque | 1.2 (1.1 - 1.4) | 1.2 (1.0 - 1.3) | 1.2 (1.0 - 1.3) | 1.2 (1.0 - 1.4) | 1.2 (1.1- 1.4) | | 1.2 (1.0- 1.4) |  |
| ^1^ All independent variables entered the same model simultaneously.  ^2^ Center for Epidemiologic Studies Depression Scale 10  ^3^ Walking for exercise | | | | | | | |  |

*Table legend: Odds ratios for each independent variable, adjusted for each significant correlate of higher self-reported energy.*

**Supplemental Figure 1: Histogram of energy level at Year Three in the Health, Aging and Body Composition Study**

*Figure legend: Self-reported energy was dichotomized at the median, as its distribution was skewed towards higher scores*
